# A Modified RMCE-Compatible Rosa26 Locus for the Expression of Transgenes from Exogenous Promoters

**DOI:** 10.1371/journal.pone.0030011

**Published:** 2012-01-13

**Authors:** Jan S. Tchorz, Thomas Suply, Iwona Ksiazek, Claudio Giachino, Dimitri Cloëtta, Claus-Peter Danzer, Thierry Doll, Andrea Isken, Marianne Lemaistre, Verdon Taylor, Bernhard Bettler, Bernd Kinzel, Matthias Mueller

**Affiliations:** 1 Novartis Institute for Biomedical Research, Developmental and Molecular Pathways, Novartis Pharma AG, Basel, Switzerland; 2 Department of Biomedicine, Institute of Physiology, University of Basel, Basel, Switzerland; 3 Max-Planck Institute for Immunobiology, Freiburg, Germany; 4 Department of Biomedical Science, Centre for Stem Cell Biology, University of Sheffield, Sheffield, United Kingdom; French National Centre for Scientific Research, France

## Abstract

Generation of gain-of-function transgenic mice by targeting the Rosa26 locus has been established as an alternative to classical transgenic mice produced by pronuclear microinjection. However, targeting transgenes to the endogenous Rosa26 promoter results in moderate ubiquitous expression and is not suitable for high expression levels. Therefore, we now generated a modified Rosa26 (modRosa26) locus that combines efficient targeted transgenesis using recombinase-mediated cassette exchange (RMCE) by Flipase (Flp-RMCE) or Cre recombinase (Cre-RMCE) with transgene expression from exogenous promoters. We silenced the endogenous Rosa26 promoter and characterized several ubiquitous (pCAG, EF1α and CMV) and tissue-specific (VeCad, αSMA) promoters in the modRosa26 locus *in vivo*. We demonstrate that the ubiquitous pCAG promoter in the modRosa26 locus now offers high transgene expression. While tissue-specific promoters were all active in their cognate tissues they additionally led to rare ectopic expression. To achieve high expression levels in a tissue-specific manner, we therefore combined Flp-RMCE for rapid ES cell targeting, the pCAG promoter for high transgene levels and Cre/LoxP conditional transgene activation using well-characterized Cre lines. Using this approach we generated a Cre/LoxP-inducible reporter mouse line with high EGFP expression levels that enables cell tracing in live cells. A second reporter line expressing luciferase permits efficient monitoring of Cre activity in live animals. Thus, targeting the modRosa26 locus by RMCE minimizes the effort required to target ES cells and generates a tool for the use exogenous promoters in combination with single-copy transgenes for predictable expression in mice.

## Introduction

Sequencing the genome of humans and rodents has provided an immense set of uncharacterized genes, and within the past decades several genetic approaches have been taken in order to address their function. Embryonic stem (ES) cells [1] are pluripotent cells [2], [3] that have served as a powerful tool to study gene functions *in vitro* and to generate knockout mice via homologous recombination [4]. In order to complement data gained from loss-of-function approaches, *in vivo* gain-of-function experiments have been carried out by generating mice overexpressing a gene of interest. Gain-of-function mouse models have been mainly generated by pronuclear microinjection [5] and random integration of the transgene into the genome. This quite often results in variable copy numbers, unpredictable expression profiles and sometimes gene silencing effects, therefore requiring extensive characterization of several independent transgenic lines [6]. Thus, insertional mutagenesis and the positional influence of endogenous genes and regulatory elements often lead to misinterpretation of the phenotypes observed [7], [8], [9]. Targeting a single-copy transgene to a specific and well-defined locus can minimize these problems and provide a predictable and reproducible expression profile.

The Rosa26 locus has been used to drive ubiquitous gene expression from the Rosa26 promoter [10]. This locus offers an open chromatin configuration in all tissues and disruption of the Rosa26 gene produces no overt phenotype, which made it one of the most commonly used genetic loci for targeted transgenesis [10], [11]. However, targeting transgenes to the endogenous Rosa26 promoter results only in moderate ubiquitous expression and is not suitable for high expression levels [12], [13], [14]. In contrast, targeting transgenes into the βactin locus yields high transgene expression levels but causes problems because heterozygous β-*actin* deletion produces phenotypes [15], [16]. Exogenous promoters targeted to the Rosa26 locus could allow high ubiquitous transgene expression or even tissue-specific expression. The chicken β-actin (pCAG) promoter targeted to the Rosa26 locus allows much higher transgene expression *in vivo* [14]. Whether other strong and ubiquitous promoters or tissue-specific promoters retain their functional properties in the Rosa26 locus is unknown.

Recent studies suggest that the Rosa26 promoter can influence transgene expression mediated by exogenous promoters inserted at this locus both *in vitro* [17] and *in vivo* [14]. The pCAG promoter in the Rosa26 locus suffers from mosaic transgene expression in multiple organs [14]. Insulator sequences have been successfully introduced into the murine hypoxanthine phosphoribosyltransferase (HPRT) locus [18] in order to shield inserted transgenes from the influence of the HPRT promoter [19], and in this case tissue-specific promoters have been shown to retain their specificity [20]. This allows for tissue-specific transgene expression using specific promoters (e.g. to generate Cre lines). However, the HPRT locus is on the X chromosome which results in random inactivation of the inserted transgene in female mice [19], [20]. Thus, it would be desirable to modify the Rosa26 locus to minimize the influence of the Rosa26 promoter on transgenes targeted to this locus.

Targeting the Rosa26 locus and other loci was mainly achieved by homologous recombination in ES cells and therefore required time-consuming and extensive screening of hundreds of ES cell clones [10], [11], [12], [13]. In contrast, recombinase-mediated cassette exchange (RMCE) using heterospecific recognition targets allows for very efficient and rapid targeted transgenesis in previously modified ES cells [15], [21]. RMCE of transgenes with exogenous promoter into a modified Rosa26 locus that contains a shielded integration site would therefore be an ideal tool for rapid generation of transgenic mice.

Here we report the generation of two ES cell lines with modified Rosa26 loci that allow for either Cre/LoxP (modRosa26^LoxP^ ES cells)- or Flp/FRT (modRosa26^FRT^ ES cells)-mediated RMCE. We shielded the integration site with a Stop sequence to facilitate the use of exogenous promoters. Using this system, several ubiquitous and tissue-specific promoters were tested *in vivo* for their utility when targeted to the modRosa26 locus. The methods presented here not only minimize the time required for successful targeting of the Rosa26 locus, but also demonstrate that the modified Rosa26 loci, in combination with exogenous promoters, represent versatile and validated tools for the generation of transgenic mouse models.

## Materials and Methods

### Statement on Animal Welfare

All experiments were carried out in accordance with authorization guidelines for the care and use of laboratory animals. Studies described in this report were performed according to Novartis animal license numbers 1022, 1331, 1943 and 2116.

### Cell culture

Mouse BALB/c-I cells [22] were cultured in ES cell medium [DMEM medium (Gibco) containing 15% fetal calf serum, 20 mM HEPES, 0.1 mM β-mercaptoethanol and 50 µg/ml penicillin/streptomycin) and 1,000 U/ml leukemia inhibitory factor on gelatin-coated culture dishes on mitomycin C-treated embryonic mouse fibroblasts. Neural stem cell cultures were prepared and maintained as previously described [23]. Briefly, telencephali were dissected from E14.5 mR26CS-EGFP/Nestin-Cre and mR26CS-EGFP control mice, the meninges were removed and the tissue was triturated in neurosphere medium consisting of DMEM/F12 (1∶1), 0.2 mg/ml L-glutamine, 1% penicillin/streptomycin, 2% B27, 2 µg/ml heparin, 20 ng/ml EGF and 10 ng/ml FGF2. Neurospheres were split after 5–6 days and plated on coverslips coated with 15 µg/ml poly-L-ornithine and 40 µg/ml laminin at a density of 200,000 cells/cm^2^. The dispersed cultures were differentiated in neurosphere medium without FGF2, EGF and heparin and fixed with 4% paraformaldehyde (PFA) after 5 days.

### Generation of the modR26^LoxP^ and modR26^FRT^ loci by homologous recombination in BALB/c ES cells

The Rosa26 locus on chromosome 6 was targeted in BALB/c-I (hereafter BALB/c ) ES cells by homologous recombination between exons 1 and 2 of the Rosa26 gene [11]. The Rosa26 homologous recombination targeting plasmid was constructed by amplifying the recombination arms using Rosa26 genomic sequences as a template (kind gift of U. Müller, FMI Basel, Switzerland). A 2.6-kb long arm (primers: sense 5′-AAAAGGTACCAATGTTCAAGCAGGACCAAA-3′ and antisense 5′-AAAAGGTACCAGATCTCTGAGTTTGAGCCC-3′) and a 1.8-kb short arm (primers: sense 5′-AAAAGCGGCCGCAAACAAATAGGATACTAGAA-3′ and antisense 5′-AAAAGAGCTCAGGCTTAAAGGCTAACCT-3′) were amplified and subcloned into a pBSIIKS+ plasmid. A synthetic Stop cassette [24] as well as an SV40 promoter-hygromycin selection cassette flanked with heterospecific Lox511/LoxP sites [25] were cloned in between the 5′ and 3′ Rosa26 homology arms resulting in the Rosa26 homologous recombination plasmid (HR modR26^LoxP^). A second Rosa26 homologous recombination plasmid (HR modR26^FRT^) was constructed as described above, but heterospecific FRT3/FRT wildtype (wt) sites [26] flanked the SV40 promoter-hygromycin selection cassette. BALB/c mouse ES cell culture was performed with primary X-ray-inactivated embryonic fibroblasts derived from DR4 mice. ES cells were transfected by electroporation using 20 µg of SacI-digested HR modR26^LoxP^ or HR modR26^FRT^ plasmid. Transfected ES cells were selected for hygromycin resistance using 0.1 mg/ml hygromycin (Roche #843 555). Ten days after transfection, 500 Hyg-resistant ES cell clones were isolated and analyzed by PCR for homologous recombination. For this purpose, ES cell DNA was extracted in 50 µl of lysis buffer [10 mM Tris-HCl (pH 8.0), 0.05% SDS, 50 µg/ml proteinase K], and diagnostic PCR was performed using 1 µl of crude ES cell extract in a total volume of 25 µl using the Qiagen Taq PCR Master Mix (primers: sense 5′-CGACTTGAGTTGCCTCAAGA-3′ and antisense 5′-TGGCTGAACTGAGCGAACA-3′). Then, 1 µl of the first reaction was used as a template for a nested PCR (primers: sense 5′-GGCAGGAAGCACTTGCTCTC-3′ and antisense 5′-ACAACAACGGCGGCTACAAC-3′), yielding a 2.1-kb fragment in positive clones (data not shown). Positive recombination events were further validated by Southern blot analysis (data not shown). We only observed 1% efficiency when homologous recombination was used to target the Rosa26 locus in BALB/c ES cells, which is in contrast to reports which show much higher recombination efficiency in 129 ES cells [14].

### Construction of the RMCE plasmids

#### Cre-RMCE plasmids

The Cre-RMCE plasmid backbone was based on a pCE-tkNeo plasmid containing heterospecific Lox511 and LoxP sites and a herpes simplex virus thymidine kinase promoter-driven neomycin resistance gene (NeoR) cassette [16]. To enable tetracycline-regulated gene expression from the cytomegalovirus (CMV) promoter, a SmaI/XhoI TRE fragment was isolated from the TRE2-puro plasmid (Clontech) and cloned in an AflIII-blunted site of pEGFP-N-1 (Invitrogen) to create pEGFP-N1-TRE. The resulting TRE-CMV-EGFP AflIII/AflII fragment was blunted before ligation into the SmaI-digested pCE-tkNeo plasmid, yielding the pCE-tkNeo-TRE-CMV-EGFP vector. To allow excision of the NeoR cassette, it was replaced by an FRT-flanked NeoR cassette in the EcoRV-digested pCE-tkNeo-TRE-CMV-EGFP plasmid to finally generate the mR26-CMV-EGFP Cre-RMCE plasmid. Next, the mR26-EF1α-EGFP plasmid was generated by isolating a 1,251-bp elongation factor 1α (EF1α) promoter fragment from the pNAS-092 plasmid [27] which was then ligated into the BglII/NsiI-digested mR26-CMV-EGFP Cre-RMCE plasmid. In addition the pCAG promoter [28] was first subcloned from a pCAGGS-eFLP [15] into the NdeI/EcorI-digested pEGFP-N1-TRE plasmid and subsequently into the NdeI/XmaI-digested mR26-CMV-EGFP, yielding the mR26-pCAG-EGFP plasmid.

#### Flp-RMCE plasmids

To clone the VeCad-Cre-Flp-RMCE plasmid, the mouse vascular endothelial cadherin promoter (VeCad) promoter [29] was amplified by Solvias AG such that the amplification product contains XbaI restriction sites at both ends. The VeCad promoter fragment was subcloned into NheI-linearized Flp-RMCE1 plasmid. Flp-RMCE1 contained FRT3 and FRTwt sites flanking a multiple cloning site and a herpes simplex virus thymidine kinase promoter-driven NeoR cassette as a group as well as an ampicillin resistance cassette. In contrast to the Cre-RMCE-based design, the NeoR cassette cannot be removed in the Flp-RMCE-based design. Finally NLS-Cre [30] was amplified and cloned into the NotI restriction site downstream of the VeCad promoter, resulting in the VeCad-Cre-Flp-RMCE plasmid. The αSMA-Cre-Flp-RMCE plasmid was cloned by amplifying the α-smooth muscle actin (αSMA) promoter [31] while introducing NheI restriction sites at both ends. The αSMA promoter fragment was then cloned into the NheI-linearized Flp-RMCE. Finally NLS-Cre was amplified and cloned into the NotI restriction site downstream of the αSMA promoter, yielding the αSMA-Cre-Flp-RMCE plasmid. The Flp-RMCE2 plasmid was based on Flp-RMCE1, containing in addition the pCAG promoter [28] and a LoxP-flanked Stop cassette [24]. The mR26CS-EGFP plasmid was constructed by cloning an AgeI/NotI-digested enhanced green fluorescent protein (EGFP) fragment from pEGFP-N1 (Invitrogen) into the AgeI/NotI-digested Flp-RMCE2. The mR26CS-Luc plasmid was generated using the Flp-RMCE3 plasmid as a backbone, which was identical to the Flp-RMCE2 plasmid but used a different LoxP-flanked Stop cassette (Stop2) that is based on the 3′-UTR of the mouse albumin gene [32]. We introduced the new Stop cassette to avoid possible recombination events that could occur when using the same cassette twice. However, to date we never observed such recombination event in any of our studies. The luciferase was amplified while introducing AgeI/NotI sites and cloned into the AgeI/NotI-digested Flp-RMCE3.

### RMCE in modRosa26^LoxP^ and modRosa26^FRT^ ES cells

**Figure 1:**
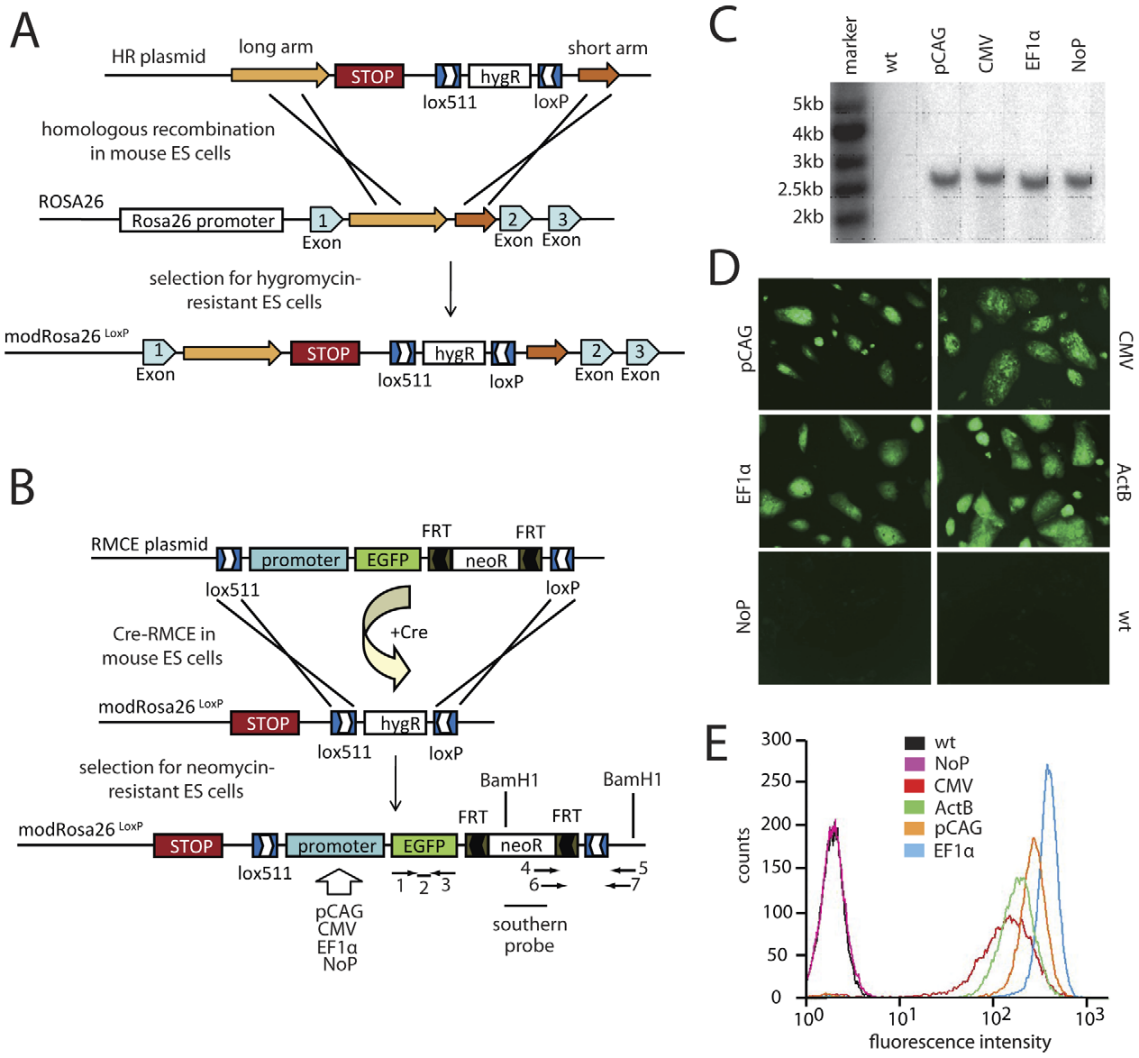
Generation of mice with a modified Rosa26 locus (modRosa26^LoxP^) and testing of different promoters. (A) Scheme depicting the generation of the modRosa26^LoxP^ in ES cells. A Stop sequence and a HygR selection cassette flanked by heterospecific LoxP sites (Lox511 and LoxP) were targeted to the Rosa26 locus between exons 1 and 2 by homologous recombination. After successful recombination, the Stop cassette is located downstream of the endogenous Rosa26 promoter. (B) Cre-RMCE into the modRosa26^LoxP^ locus. In the Cre-RMCE targeting plasmid, a promoter, EGFP and an FRT-flanked neomycin selection cassette (NeoR) were flanked by heterospecific LoxP sites (Lox511 and LoxP) as a group. Cre-RMCE was used to replace the HygR in the modRosa26^LoxP^ ES cells with the Lox511/LoxP-flanked sequence in the RMCE targeting plasmid. A pCAG, CMV or EF1α promoter driving EGFP was introduced. Insertion of EGFP without any promoter (NoP) controls for functional shielding of the integration site from the endogenous Rosa26 promoter. Binding regions for TaqMan genotyping primers (1, 3) and probe (2), primers for checking integration into the modRosa26^LoxP^ locus (4–7) and the Southern hybridization probe, as well as the BamHI sites used for Southern blot analysis are indicated. (C) Southern blot analysis on genomic DNA-derived ES cell lines shows specific integration into the modRosa26^LoxP^ locus. A 2.4-kb BamHI fragment was detected using a Neo probe. mR26-pCAG-EGFP, mR26-CMV-EGFP, mR26-EF1α-EGFP and mR26-NoP-EGFP ES cells show successful targeting of the modRosa26^LoxP^ locus, without additional integrations at random sites. Wt ES cells show no signal. (D) Modified ES cells showing strong EGFP fluorescence. The pCAG, EF1α and CMV promoters drive strong EGFP expression in the modRosa26^LoxP^ in vitro. ES cells without promoter but with EGFP inserted into the modRosa26^LoxP^ locus do not show EGFP fluorescence, indicating functional shielding of the integration site from the endogenous Rosa26 promoter. (E) FACS analysis in mR26-NoP-EGFP ES cells showed no EGFP fluorescence when compared to wt ES cells. mR26-EF1α-EGFP ES cells showed the highest EGFP fluorescence, followed by mR26-pCAG-EGFP, ActB and mR26-CMV-EGFP ES cells.

A total of 0.5×10^6^ ES cells were treated with Effectene (Qiagen, Chatsworth, CA, USA) and cotransfected with 0.8 µg of pMC-Cre and 0.2 µg of Cre-RMCE plasmid according to the manufacturer's protocol and subsequently selected for G418 resistance. Cre-mediated recombination into the modRosa26^LoxP^ locus was confirmed by PCR and Southern hybridization (data not shown and Figure 1C). DNA for PCR and Southern blot was isolated by pelleting ES cells using centrifugation and digestion in lysis buffer (ddH_2_O, 0.05% SDS, 1 mM TrisHCl and 25 µg/ml proteinase K) overnight at 55°C. The 1∶10 diluted digested samples were then used for PCR, whereby the first PCR (primers: sense 5′-AGCAGCCGATTGTCTGTTGT-3′, antisense 5′-TGTGTGTATTCCTGGCTATCC-3′) was used as a template for a nested PCR (primers: sense 5′-TCATAGCCGAATAGCCTCTC-3′, antisense 5′-TGATGTGTAGACCAGGCTGG-3′) in order to amplify a 594-bp fragment (data not shown). For Southern blot analysis, genomic DNA was digested with BamHI overnight, run on a 1% agarose gel and blotted on a nylon membrane (Hybond N+, Amersham). Hybridization was performed overnight at 65°C using a 1.2-kb hybridization probe derived from the NeoR gene labeled with ^32^P (Rediprime II Random prime labeling kit, Amersham), detecting a 2.4-kb BamHI fragment in mice with the targeted modR26^LoxP^ locus. After washing, the membrane was exposed to a Kodak BioMax MS film. For detection of Flp-mediated RMCE, ES were treated as described above and cotransfected with 0.8 µg of pCAG-flpe [15] and 0.2 µg of flp-RMCE plasmid. Flp-mediated recombination into the modRosa26^FRT^ locus was confirmed by TaqMan PCR (sense primer 5′-ATATCCGCGGTGGAGATCAA-3′, antisense primer 5′-TAGACCAGGCTGGGCTAAA-3′, probe 5′-VIC-CGGTACCAGATCTC-MGB-3′) and Southern blot analysis (data not shown).

### Generation, breeding and genotyping of transgenic mice

ES cells with cassette exchange events in the modRosa26^LoxP^ or modRosa26^FRT^ locus and preserved karyotypes were used for blastocyst injection as described elsewhere [16]. Chimeric mice were mated with BALB/c mice. Germline transmission was observed for all lines targeted to both the modR26^LoxP^ (mR26-CMV-EGFP, mR26-EF1α-EGFP, mR26-pCAG-EGFP) and the modRosa26^FRT^ locus (mR26-VeCad-Cre, mR26-αSMA-Cre, mR26CS-EGFP and mR26CS-LUC). Heterozygous transgenic mice with a targeted modRosa26^LoxP^ locus were used for EGFP fluorescence and immunohistochemistry analysis. Genotyping for mice with successful Cre-RMCE targeting of the modRosa26^LoxP^ locus was performed by TaqMan PCR. In brief, small tail biopsies were digested in proteinase K-containing lysis buffer overnight at 55°C. The 1∶10 diluted digested samples were then genotyped by TaqMan PCR for NeoR (sense primer 5′-GCCCGGTTCTTTTTGTCAAG-3′, antisense primer 5′-GCCTCGTCCTGCAGTTCATT-3′ , probe 5′-FAM-CCGACCTGTCCGGTGCCC-TAMRA-3′) or EGFP (sense primer 5′-ACAGCTCGTCCATGCCGA-3′, antisense primer 5′-TCACATGGTCCTGCTGGAGT-3′, probe 5′-FAM-TGATCCCGGCGGCGGTCA-TAMRA-3′). Heterozygous transgenic mice with the targeted modR26^FRT^ locus were crossed with CMV-Cre [33], Nestin-Cre [34], Myf5-Cre [35] and Glast-CreER^T2^ [36], [37] mice for conditional Cre-mediated transgene activation. Genotyping was performed by TaqMan PCR for the Flp-RMCE-targeted modRosa26^FRT^ locus (sense primer 5′-ATATCCGCGGTGGAGATCAA-3′, antisense primer 5′-TAGACCAGGCTGGGCTAAA-3′, probe 5′-VIC-CGGTACCAGATCTC-MGB-3′), Cre (sense primer 5′-GCCGCGCGAGATATGG-3′, antisense primer 5′-GCCACCAGCTTGCATGATC-3′, probe 5′-FAM-CCGCGCTGGAGTTTCAATACCGG-TAMRA-3′) and luciferase (sense primer 5′-GACGGAAAAAGAGATCGTGGAT-3′, antisense primer 5′-CAACTCCTCCGCGCAACT-3′, probe 5′-FAM-CGCCAGTCAAGTAACAACCGCGAAA-TAMRA-3′). All mice with the targeted modRosa26^LoxP^ or modRosa26^FRT^ locus were born at a normal Mendelian ratio and showed no overt phenotype. All animals had unrestricted access to water and food. Protocols, handling and care of the mice conformed to the Swiss federal law for animal protection.

### FACS analysis and EGFP quantification

ES cell clones with EGFP driven by different promoters after Cre-RMCE targeting to the modR26^LoxP^ locus were harvested by trypsinization, and the EGFP fluorescence from 10^6^ cells was determined by FACS Analysis (BD FACScalibur). Mice were euthanized with CO_2_, and selected organs (salivary glands, heart, pancreas, liver, kidney, fat, muscle, brain and testis) were excised, rinsed with ice-cold PBS and placed on ice. Whole-organ pictures were taken at low magnification using a Zeiss Axiovert 25 binocular fluorescence microscope (Zeiss). Organ homogenates were prepared in PBS containing protease inhibitors (Roche) and subsequently analyzed for EGFP fluorescence using a 96-well fluorescence reader (Wallac 1420 at 485 nm/535 nm, 1.0 s). Background fluorescence levels for each organ were determined using BALB/c wt littermates and subtracted from the relative fluorescence values for each organ (n≥5 mice per genotype).

### Immunohistochemistry

Mice were sacrificed by cervical dislocation, and organs were removed. Samples for protein, DNA and RNA analysis were immediately frozen at −80°C. Samples for histological examination were fixed overnight with 4% PFA in phosphate-buffered saline (PBS) at 4°C and frozen in OCT (TissueTEK). Then, 10-µm-thick cryostat sections were mounted, washed in PBS and incubated for 1 h in blocking solution (2% BSA and 0.2% Triton X-100 in PBS), followed by overnight incubation at 4°C in blocking solution containing primary antibodies, Alexa-594-conjugated wheat germ agglutinin (W11262, Molecular Probes; 1∶1,000) or biotinylated *Dolichos biflorus* agglutinin (Vector Laboratories, Burlingame, CA, USA; 1∶100). Alternatively, mice were deeply anaesthetized by injection of a ketamine/xylazine/flunitrazepam solution (150 mg, 7.5 and 0.6 mg/kg body weight, respectively) and perfused with ice-cold 0.9% saline solution followed by ice-cold 4% PFA solution in 0.1 M phosphate buffer. Brains were post-fixed with 4% PFA overnight, washed in phosphate buffer, cryoprotected in a 30% sucrose solution in 0.1 M phosphate buffer for 48 h, embedded and frozen in OCT (TissueTEK). Free-floating coronal sections (30 µm) were collected in multi-well dishes and stored at −20°C in antifreeze solution until use. For immunostaining, sections were incubated overnight at 4°C with the primary antibody diluted in blocking solution of 2% normal donkey serum (Jackson ImmunoResearch) and 0.5% Triton X-100 in PBS. The primary antibodies used were rabbit anti-hepatocyte nuclear factor 4α (HNF4α) (H-171, Santa Cruz Biotechnology, Santa Cruz, CA, USA; 1∶100), mouse anti-NeuN (MAB377, Chemicon; 1∶500), rabbit anti-GFAP (ZO334, Dako; 1∶1,000), mouse anti-NeuN (Sigma; 1∶800), rabbit anti-BLBP (Chemicon; 1∶1,000), mouse anti-GFAP (Chemicon; 1∶1,000) and rabbit anti-MAP2 (Chemicon; 1∶500). After rinsing in PBS, sections were incubated in blocking solution containing secondary antibodies for 1 h at room temperature. The secondary antibodies used were Cy3-conjugated donkey anti-rabbit, Cy3-conjugated donkey anti-mouse, Cy5-conjugated donkey anti-rabbit, Cy5-conjugated donkey anti-mouse, Cy5-conjugated donkey anti-sheep and streptavidin-Alexa 647 (Jackson ImmunoResearch, UK; 1∶500). Immunofluorescence sections were imaged using a Leica DMI6000 fluorescence microscope or a Zeiss LSM510 confocal microscope. For diaminobenzidine (DAB) staining, tissue was fixed in 10% buffered formalin for 48 h and embedded in paraffin using a standard procedure. Then, 3-µm sections were cut using a microtome and rehydrated, and endogenous peroxidase activity was quenched with 0.5% H_2_O_2_ in methanol for 20 min, followed by washing with Tris-buffered saline containing 0.5 M Tris and 0.9% NaCl, pH 7.6 (TBS). Sections were then blocked with 10% goat serum in TBS for 20 min and incubated with rabbit anti-EGFP antibodies (589, MBL; 1∶500) in 1% goat serum containing TBS overnight at 4°C. Immunostaining was completed using the Vectastain ABC Kit (PK-6101, Vector Laboratories) according to the manual and followed by 15-min incubation with DAB (Dako) and counterstaining with hematoxylin. Sections were analyzed with an Axio ImagerZ1 microscope equipped with an AxioCam MRc Rev3 color camera.

### Luciferase reporter gene assay

Mice were deeply anesthetized with pentobarbital (10 mg/kg body weight), and the fur was shaved. Firefly D-Luciferin (Caliper Life Sciences) was dissolved in PBS then filtered, and 10 ml/kg body weight was injected i.p. into the mice. Luminescence was measured 10 min after Firefly D-Luciferin injection using a Xenogen camera (Living Image 2.5, Caliper Life Sciences) and Living Image Software. To determine luciferase activity in organ homogenates, mice were sacrificed by cervical dislocation and organs were removed and placed on ice. Organs were homogenized by brief sonication in ice-cold PBS containing protease inhibitors (Roche). Then, 100 µl per well was transferred into 96-well plates, and 10 µl of Luciferin (Bright Glo Assay Reagent, Promega) was added to each sample. Samples were measured using a 96-well luminescence reader (Wallac 1420).

### CreER^T2^ induction by tamoxifen

In order to specifically recombine adult neural progenitors and astrocytes in mR26CS-EGFP and mR26CS-N2ICD mice, we used GLASTCreER^T2^ mice [36], [37]. Adult mice between 8 and 12 weeks of age were used for the experiments. Stock solutions of tamoxifen (Sigma) were prepared at a concentration of 20 mg/ml in corn oil (Sigma). Mice were injected i.p. with tamoxifen once per day for 10 consecutive days at a dose of 2 mg/day. Animals were sacrificed 21 days after the last injection, and the brains were prepared for immunohistochemistry as described above.

## Results

### RMCE into the modRosa26^LoxP^ locus for rapid generation of transgenic ES cells

To facilitate and accelerate gene targeting to a defined locus, we modified the well-defined Rosa26 locus for fast, easy and specific integration of various transgenic constructs in mouse BALB/c ES cells. By means of homologous recombination, we introduced a cassette that enables site-directed RMCE mediated by Cre recombinase (Cre-RMCE) (Figure 1A). ES cells with a modified Rosa26 locus harboring heterospecific LoxP sites (modRosa26^LoxP^ ES cells) were then used for Cre-RMCE, resulting in very efficient site-directed integration of the Lox511/LoxP-flanked sequence of the Cre-RMCE plasmid into the modRosa26^LoxP^ locus (Figure 1B). Recombination events were identified by a shift from hygromycin resistance to G418 resistance and confirmed by PCR (data not shown) and Southern blot analysis (Figure 1C). Site-directed Cre-RMCE into the modRosa26^LoxP^ locus occurred in 95±4% of all G418-resistant/hygromycin-sensitive clones (n = 5 independent RMCEs). Therefore, we drastically minimized the time and effort required to target transgenes to this locus when compared to other homologous recombination strategies.

### Comparison of different ubiquitous promoters in the modRosa26^LoxP^ locus *in vitro*

For functional testing of transgene expression from the modRosa26^LoxP^ locus using exogenous promoters, we introduced three different ubiquitous promoters driving an EGFP reporter gene by Cre-RMCE into modRosa26^LoxP^ ES cells. Since the Rosa26 promoter offers only moderate expression levels [14], we aimed to find a strong and ubiquitous promoter that can be used in the modRosa26^LoxP^ locus for high-level transgene expression. The promoters tested were the chicken β-actin promoter (pCAG) [28], the elongation factor 1α promoter (EF1α) [38] and the cytomegalovirus (CMV) promoter [39]. As a reference control we used ES cells, that have EGFP targeted to the β-actin locus (ActB ES cells) and have been shown to induce much higher expression levels than the Rosa26 promoter [16]. BALB/c wt ES cells and ES cells with EGFP targeted to the modRosa26^LoxP^ locus without any promoter (mR26-NoP-EGFP) served as negative controls. Transgenic ES cells driving EGFP from the pCAG (mR26-pCAG-EGFP), EF1α (mR26-EF1α-EGFP) or CMV (mR26-CMV-EGFP) promoter showed strong EGFP fluorescence, comparable to ACTB ES cells (Figure 1D). As in wt ES cells, no EGFP fluorescence could be detected when no promoter was present (modR26-NoP-EGFP) (Figure 1D). These results were confirmed by FACS analysis for EGFP fluorescence intensity using these ES cells. Wt and mR26-NoP-EGFP ES cells showed no EGFP fluorescence, whereas mR26-EF1α-EGFP ES cells showed the highest EGFP fluorescence, followed by ES cells with the pCAG or CMV promoter (Figure 1E). The absence of EGFP fluorescence in mR26-NoP-EGFP ES cells showed that the endogenous Rosa26 promoter was functionally silenced by the inserted Stop sequence.

### Comparison of different ubiquitous promoters in the modRosa26^LoxP^ locus *in vivo*

**Figure 2:**
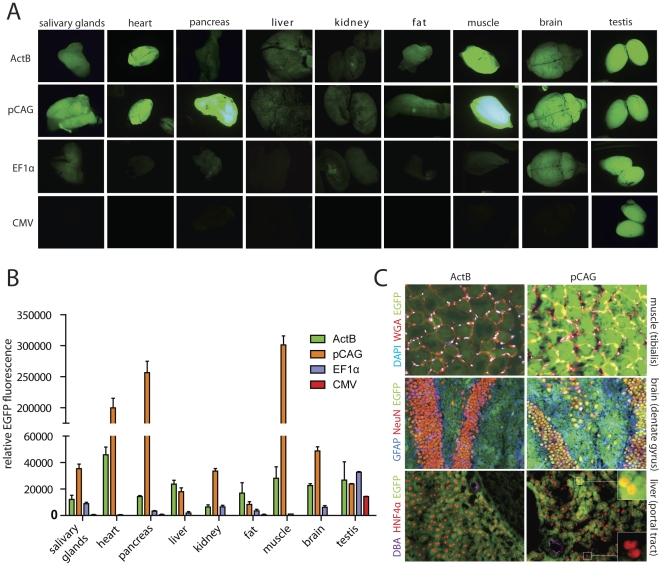
Comparison of different ubiquitous promoters in the modRosa26^LoxP^ locus. (A) Organs from transgenic mice showing different intensity of overall EGFP fluorescence under low-magnification microscopy, using the same conditions. ActB mice were used as controls for high EGFP expression levels. The pCAG promoter shows the highest overall fluorescence. The CMV promoter showed strong activity only in testis. (B) EGFP fluorescence was quantified in organ homogenates from mR26-pCAG-EGFP, mR26-EF1α-EGFP, mR26-CMV-EGFP and ActB mice. In most organs, EGFP fluorescence was highest in mR26-pCAG-EGFP mice, except fat tissue where ActB mice showed higher levels. Heart, pancreas and muscle showed extremely high levels of EGFP fluorescence. Background fluorescence determined in organ homogenates from wt mice was subtracted (n≥3 mice per genotype). Values are shown as mean ± SEM. (C) Exemplary pictures from cryosections showing strong and ubiquitous EGFP expression in muscle (costained with wheat germ agglutinin (WGA) for muscle fiber wall and DAPI) and brain (hippocampus, costained with NeuN for neurons and GFAP for astrocytes). Mosaic EGFP fluorescence was detected in livers of mR26-pCAG-EGFP mice (costained with HNF4α for hepatocytes and DBA for bile ducts). Magnified insets show both EGFP+ and EGFP- hepatocytes.

Since the CMV, EF1α and pCAG promoters proved to allow high-level transgene expression *in vitro* when targeted to the modRosa26^LoxP^ locus, we then tested their activity in this locus *in vivo*. For this purpose, we generated transgenic mice using the mR26-pCAG-EGFP, mR26-EF1α-EGFP and mR26-CMV-EGFP ES cells. After blastocyst injection, highly chimeric offspring were mated with BALB/c mice, and germline transmission was observed for all lines generated within the first litter. All mR26-pCAG-EGFP, mR26-EF1α-EGFP and mR26-CMV-EGFP mice were born at a normal Mendelian ratio, were fertile and did show any overt phenotype. Selected organs (salivary glands, heart, pancreas, liver, kidney, fat, muscle, brain and testis) from 6- to 8-week-old mice were analyzed for EGFP expression (Figure 2A). EGFP fluorescence in organ homogenates was quantified, and the fluorescence levels driven from the pCAG, EF1α and CMV promoters were compared to those in ActB mice [16] (Figure 2B). Since these mice express EGFP from the endogenous β-actin locus, they offer high EGFP expression but the heterozygous loss of β-actin results in an overt phenotype and homozygous loss of β-actin is embryonically lethal [16]. Therefore we aimed to find a promoter which offers a similarly high expression level in the Rosa26 locus but does not have such disadvantages. Surprisingly, mR26-CMV-EGFP mice did not show ubiquitous EGFP expression *in vivo*, in contrast to the high EGFP expression seen in undifferentiated mR26-CMV-EGFP ES cells *in vitro*. Relatively high EGFP fluorescence was only found in testis, and some mosaic staining was present in heart and pancreas (Figure 2A, 2B, data not shown). The mR26-EF1α-EGFP mice showed moderate EGFP fluorescence levels in all organs analyzed and strong levels in testis, although all levels were lower than in ActB mice. In contrast, mR26-pCAG-EGFP mice showed much higher levels of EGFP fluorescence than ActB mice in most organs. Only in fat tissue ActB mice showed higher EGFP fluorescence than mR26-pCAG-EGFP mice, while liver and testis showed comparable EGFP fluorescence levels. Heart, pancreas and muscle showed extremely high EGFP fluorescence in mR26-pCAG-EGFP mice, 10 times higher than that seen in the same organs of ActB mice (Figure 2B). Histological analysis using cryosections prepared from muscle, brain and liver (Figure 2C) and other organs (kidney, lung and heart; data not shown) of mR26-pCAG-EGFP mice showed an overall higher level of EGFP fluorescence (visible without EGFP antibody staining) than that seen in comparable sections prepared from ActB mice. However, in liver sections from mR26-pCAG-EGFP mice EGFP fluorescence was highly mosaic. While many HNF4α-positive hepatocytes and *Dolichos biflorus* agglutinin-positive bile ducts showed high EGFP fluorescence, some hepatocytes were EGFP-negative (Figure 2C). In skeletal muscle sections the EGFP fluorescence was extremely high in mR26-pCAG-EGFP mice, shown by costaining with the muscle membrane marker wheat germ agglutinin and DAPI. In the brain, NeuN- (neurons) and GFAP-positive cells (astrocytes and neural stem cells) are also EGFP-positive. DAB staining on paraffin sections from liver, kidney, brain, lung and heart using EGFP antibodies further confirmed these results (Figure S1). In summary, the pCAG promoter offers very strong and ubiquitous transgene expression in our modified Rosa26 locus. However, in liver it shows a mosaic expression pattern (Figure 2C, Figure S1). The EF1α promoter can be useful if moderate transgene levels are required. The CMV promoter is not suitable for reliable transgene expression from the modRosa26^LoxP^ locus, possibly due to silencing effects that have been reported previously [40].

### Tissue-specific promoters in the modRosa26^LoxP^ locus

**Figure 3:**
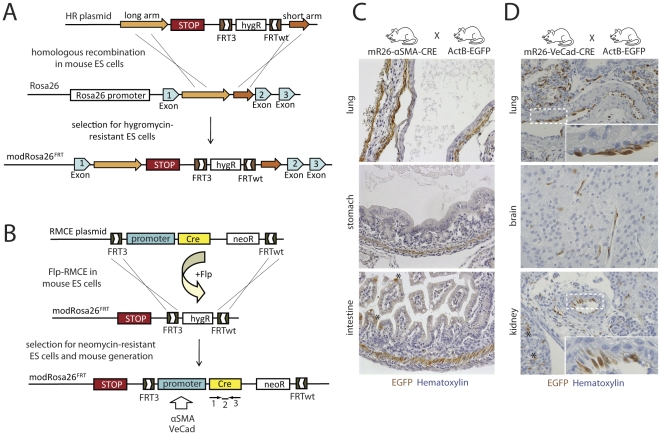
Tissue-restricted gene expression from the modRosa26^FRT^ locus. A) Scheme depicting the generation of the modRosa26^FRT^ locus in ES cells. A Stop sequence and a HygR selection cassette flanked by heterospecific FRT sites (FRT3 and FRTwt) were targeted to the Rosa26 locus between exons 1 and 2 by homologous recombination. After successful recombination, the Stop cassette is located downstream of the endogenous Rosa26 promoter (B) Flp-RMCE was performed by introducing the VeCad or αSMA promoter driving Cre recombinase into modRosa26^FRT^ ES cells, which were then used to generate transgenic mice (mR26-VeCad-Cre or mR26-αSMA-Cre mice). The primers and probe for genotyping are indicated (1–3). Both lines were crossed with ActB-EGFP reporter mice to monitor Cre expression, yielding mR26-VeCad-Cre/ActB-EGFP and mR26-αSMA-Cre/ActB-EGFP mice. (C) Immunostaining for EGFP in mR26-αSMA-Cre/ActB-EGFP mice shows αSMA promoter activity almost exclusively in smooth muscle cells, as seen in lung, stomach and intestine. Few EGFP stained intestinal epithelial cells indicate ectopic SMA promoter activity (asterisks) (D) Immunostaining for EGFP in mR26-VeCad-Cre/ActB-EGFP mice reveals VeCad promoter activity mostly restricted to endothelial cells, as seen in lung, brain and kidney. Some EGFP stained epithelial cells in kidney also indicate ectopic VeCad-Cre expression (asterisks).

We next tested whether tissue-specific promoters inserted into the modRosa26^LoxP^ can be used to drive transgene expression in defined cell types *in vivo*. In order to gain sensitivity in monitoring promoter specificity, we generated mice using tissue-specific promoters to drive the gene encoding for Cre recombinase. In combination with an EGFP reporter system, we were able to detect even low amounts of Cre expression throughout development. Since the Cre/LoxP system is required for conditional EGFP activation in ActB-EGFP reporter mice [15], we used Flipase-mediated RMCE (Flp-RMCE) for rapid and efficient targeting of the Rosa26 locus. Therefore, we generated the modRosa26^FRT^ locus by introducing heterospecific FRT sites (FRT3/FRTwt) in BALB/c ES cells in a manner analogous to that described above for the modRosa26^LoxP^ locus (Figure 3A). The resulting modRosa26^FRT^ ES cells were used for Flp-RMCE, introducing the vascular endothelial cadherin (VeCad) [29] and alpha smooth muscle actin (αSMA) [31] promoters driving Cre (Figure 3B). The resulting mice (mR26-VeCad-Cre, mR26-αSMA-Cre) were crossed with ActB-EGFP reporter mice [15], and the double transgenic offspring (mR26-VeCad-Cre/ActB-EGFP, mR26-αSMA-Cre/ActB-EGFP) were analyzed for EGFP expression by immunohistochemistry. Immunostaining in mR26-αSMA-Cre/ActB-EGFP mice using EGFP antibodies showed specific staining of the smooth muscle cells in vessel and gut walls in lung, stomach and intestine (Figure 3C). In addition, staining was observed in cardiac muscle (data not shown), due to the transient activity of the αSMA promoter in heart during development [41]. However, few epithelial cells of the intestinal villi were also EGFP positive, indicating rare ectopic αSMA promoter activity (Figure 3C). In mR26-VeCad-Cre/ActB-EGFP mice, EGFP staining was almost exclusively present in endothelial cells, as seen in lung, brain and kidney while neighboring muscle cells and other cell types were EGFP negative. However, few EGFP positive epithelial cells in kidney also indicate ectopic VeCad promoter activity (Figure 3C). Since low amounts of Cre expression during development or in adult mice suffice to induce EGFP expression in mR26-αSMA-Cre/ActB-EGFP or mR26-VeCad-Cre/ActB-EGFP mice, our system is very sensitive for the detection of nonspecific Cre expression. While αSMA and VeCad promoters largely retained their specificity, two different Col1a1 promoters [42] used to drive Cre expression in our system entirely lost their specificity, resulting in ubiquitous EGFP expression (data not shown). Since tissue-specific activity of promoters targeted to the modRosa26^FRT^ locus seems depend on the promoters used, this approach requires careful characterization of the individual models generated.

### Combined Flp/FRT-mediated RMCE into a modified Rosa26 locus (modRosa26^FRT^) and Cre/LoxP-mediated transgene activation

**Figure 4:**
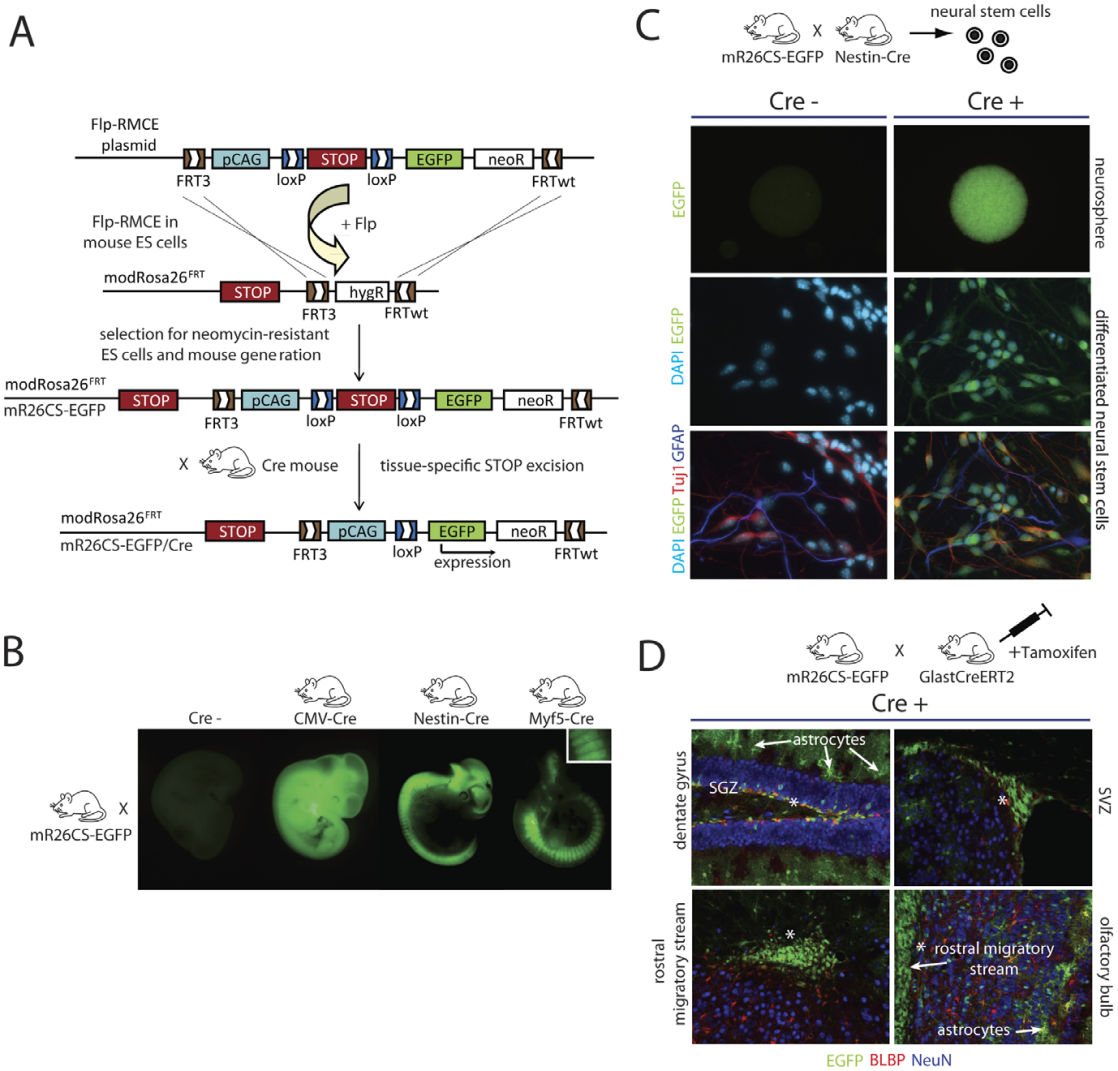
Generation of the modRosa26^FRT^ locus and a reporter strain for strong EGFP expression. (A) Flp-RMCE into the modRosa26^FRT^ locus, replacing the HygR selection cassette in the modRosa26^FRT^ ES cells with an FRT/FRTwt-flanked sequence in the Flp-RMCE targeting plasmid. The Flp-RMCE targeting plasmid contains the pCAG promoter followed by a LoxP-flanked (floxed) STOP cassette, the EGFP cDNA and a NeoR cassette, flanked as a group by FRT/FRTwt sites. Successfully targeted ES cells were used to generate mR26-CS-EGFP mice. After crossing mR26-CS-EGFP mice with several Cre mice, ubiquitous or tissue-restricted EGFP reporter expression could be obtained. (B) mR26CS-EGFP/CMV-Cre E12.5 embryos show ubiquitous EGFP expression, while mR26CS-EGFP/Nestin-Cre mice show EGFP expression restricted to the brain and neural tube. EGFP expression in E10.5 mR26CS-EGFP/Myf5-Cre embryos was restricted to the somites (magnified inset), limbs and parts of the brain. (C) Neural stem cells isolated from E14.5 mR26CS-EGFP/Nestin-Cre mice form neurospheres with ubiquitous and strong EGFP fluorescence, while mR26CS-EGFP mice show no fluorescence. These cells were subsequently differentiated (lower panels), showing strong EGFP fluorescence in mR26CS-EGFP/Nestin-Cre-derived cells (counterstained with DAPI, the neuronal marker Tuj1 and the glial marker GFAP). (D) Adult mR26CS-EGFP/Glast-CreERT2 mice show strong and specific EGFP fluorescence in astrocytes and in the adult neural stem cell niche (asterisks) upon tamoxifen administration. EGFP+ adult neural stem cells are present in the subgranular zone (SGZ) of the dentate gyrus and the subventricular zone (SVZ). Recombined EGFP+ cells from the SVZ can be traced through the rostral migratory stream into the olfactory bulb. NeuN stains for mature neurons (blue) and BLBP for adult neural stem cells and astrocytes (red). Note: no EGFP-antibody staining was used in C–E, since the mR26CS-EGFP reporter mouse offers very high EGFP expression levels which can be easily detected without any staining.

Another possibility to achieve tissue-specific transgene expression is Cre/LoxP-mediated transgene activation using tissue-specific Cre lines. In this system, a LoxP-flanked Stop cassette is placed between the promoter and the transgene, which silences its transcription. Tissue-specific Cre activity leads to the excision of the Stop cassette and enables specific transgene expression. We have shown that the pCAG promoter allows high transgene expression from the modRosa26^LoxP^ locus after Cre-RMCE. Now, we combined Flp-RMCE with Cre/LoxP conditional transgene activation. Therefore, we performed Flp-RMCE into modRosa26^FRT^ ES cells, introducing the pCAG promoter, a LoxP flanked Stop cassette [24] and the EGFP reporter gene, which were then used to generate mR26CS-EGFP mice. By crossing mR26CS-EGFP mice with different Cre lines, tissue-specific and high EGFP expression can be achieved (Figure 4A). We first crossed mR26CS-EGFP mice with CMV-Cre full deleter mice [33], yielding mR26CD-EGFP/CMV-Cre mice and Stop excision in all tissues and thus ubiquitous EGFP expression in E12.5 embryos (Figure 4B). Next, Nestin-Cre mice [34] were crossed with mR26CS-EGFP mice to activate EGFP expression exclusively in the brain and neural tube in mR26CS-EGFP/Nestin-Cre embryos (Figure 4B). E10.5 mR26CS-EGFP/Myf5-Cre embryos resulting from mR26CS-EGFP mice crossed with Myf5-Cre mice [43] show specific EGFP expression in the somites, limbs and parts of the brain, as expected (Figure 4B). Single transgenic mR26CS-EGFP mice showed no EGFP expression, demonstrating that the Stop cassette efficiently shields the EGFP gene from the pCAG promoter (Figure 4B).

Neural stem cells isolated from mR26CS-EGFP/Nestin-Cre mice formed neurospheres and displayed high EGFP fluorescence, allowing easy tracing of recombined cells without further staining. Staining for GFAP and Tuj1 in differentiated neural stem cells showed strong EGFP expression in both astrocytes (GFAP+) and neurons (Tuj1+) (Figure 4C). In order to test conditional EGFP expression in adult mR26CS-EGFP mice, we crossed them with GlastCre-ER^T2^ mice [36], [37], allowing specific recombination within astrocytes and the neural stem cell niche upon tamoxifen injection. Tamoxifen-injected mR26CS-EGFP/GlastCre-ER^T2^ mice showed strong and specific EGFP expression in the subgranular zone of the dentate gyrus and the subventricular zone, where the adult neural stem cells reside, and in astrocytes (Figure 4D). Importantly, no staining for EGFP was required to trace recombined cells. Our mR26CS-EGFP mice can therefore serve as a reporter line to trace live recombined cells (e.g. for electrophysiology). Furthermore, the specificity of Cre mice can be easily checked by analyzing tissue under a fluorescence microscope after crossing them with mR26CS-EGFP mice.

**Figure 5:**
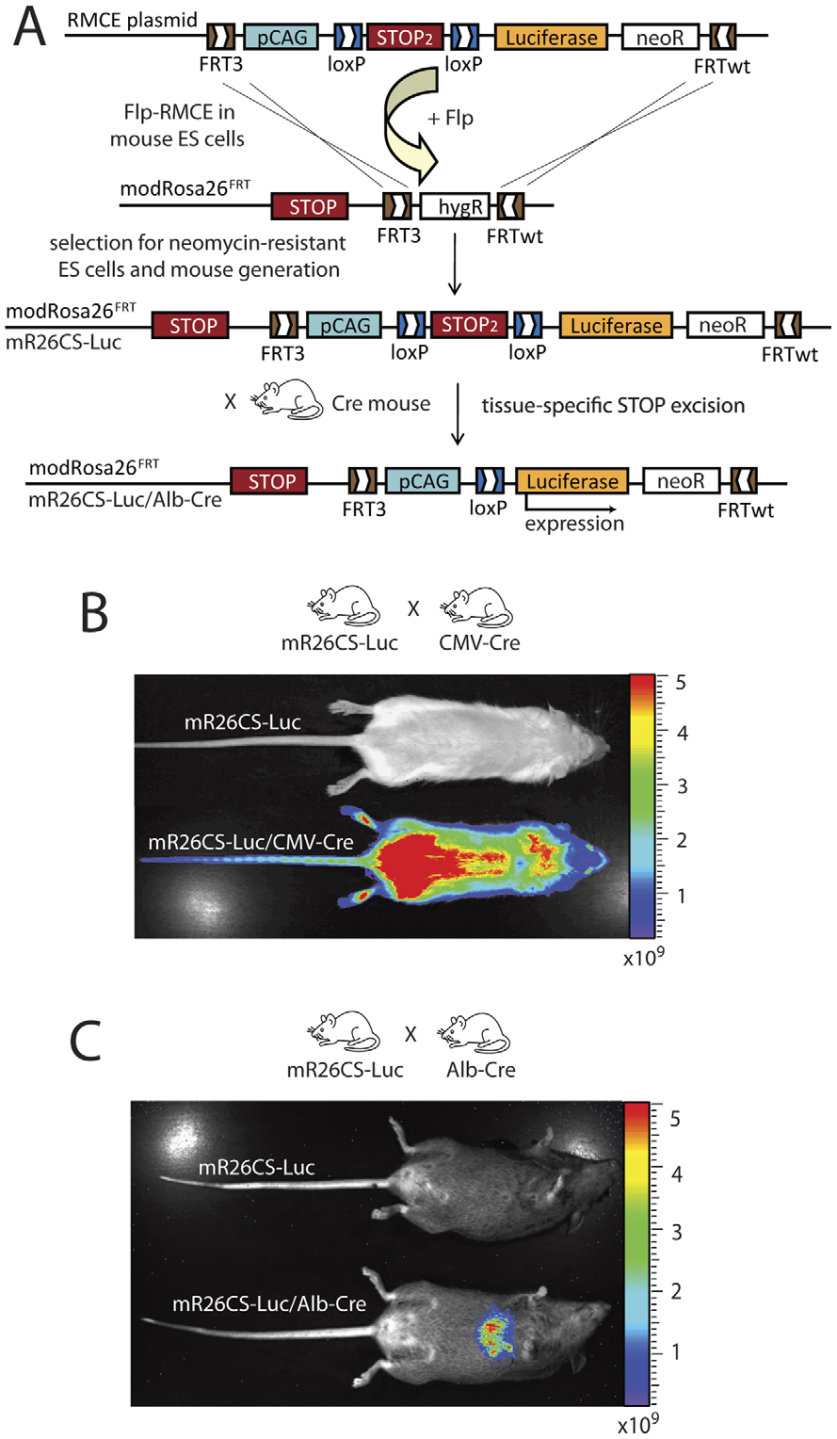
Generation of luciferase reporter mice (mR26CS-Luc). (A) Flp-RMCE into the modRosa26^FRT^ locus was performed, replacing the HygR in the modRosa26^FRT^ ES cells with the FRT/FRTwt-flanked sequence from the Flp-RMCE targeting plasmid. The Flp-RMCE targeting plasmid contains the pCAG promoter followed by a LoxP-flanked (floxed) STOP cassette (STOP2), the luciferase cDNA and a NeoR cassette, flanked as a group by FRT3/FRTwt sites. Successfully targeted ES cells were used to generate mR26CS-Luc mice. After crossing mR26CS-Luc mice with Cre mice, ubiquitous or tissue-restricted luciferase reporter expression can be obtained to monitor reporter expression in living mice upon Luciferin injection. Using Xenogen imaging, adult mR26CS-Luc/CMV-Cre mice show ubiquitous luciferase activity throughout the body (B), while mR26CS-Luc/AlbCre mice show luciferase activity restricted to the liver upon Luciferin injection (C). Luciferin-injected mR26SC-Luc control mice never showed luciferase activity.

To further improve detection of recombination events, monitoring live animals would be desirable. For this purpose we performed Flp-RMCE into modR26^FRT^ ES cells, introducing the pCAG promoter, a LoxP-flanked Stop cassette and luciferase (Luc) [44] cDNA (Figure 5A). Mice generated from positive recombined mR26CS-Luc ES cells were crossed with CMV-Cre mice to induce ubiquitous luciferase expression. Upon injection of Luciferin, mR26CS-Luc/CMV-Cre mice were imaged using a Xenogen camera and showed a strong luminescence signal throughout the body, while no signal was observed in single transgenic mR26CS-Luc control mice (Figure 5B). Next we crossed our mR26CS-Luc mice with Albumin-Cre mice (Alb-Cre) [45], allowing liver-specific luciferase expression. Tissue-specific luminescence was observed after Luciferin injection in mR26CS-Luc/Alb-Cre mice, whereas no signal was present in other tissue or in mR26CS-Luc controls (Figure 5C). Thus, mR26CS-Luc could serve as a new reporter line for rapid screening of newly generated Cre lines, since recombination events can be monitored easily in living mice.

## Discussion

The aim of this study was to improve the generation of gain-of-function ES cell lines and mouse models by facilitating the use of exogenous promoters in the Rosa26 locus and accelerating the generation of mutant ES cells using RMCE. We therefore generated two ES cell lines with modified Rosa26 loci by introducing either heterospecific Lox511/LoxP sites (modRosa26^LoxP^ ES cells) or FRT3/FRT sites (modRosa26^FRT^ ES cells). ModRosa26^LoxP^ ES cells allow for RMCE using the Cre/LoxP system, while modRosa26^FRT^ ES cells can be used for RMCE using the Flp/FRT system. All transgenic ES cell lines were successfully used to generate highly chimeric transgenic mice that showed germline transmission within the first litter. Compared to homologous recombination, RMCE into the modRosa26 locus dramatically increased the targeting efficacy and therefore minimizes time, effort and costs for the generation of transgenic ES cells and mice thereof. Most previous studies used the endogenous Rosa26 promoter to achieve ubiquitous gene expression *in vitro* and *in vivo* at moderate levels [10], [11], [12], [13]. However, the pCAG promoter targeted to the Rosa26 locus offers 8- to 10-fold stronger transgene expression when compared to the Rosa26 promoter [14]. Whether other ubiquitous promoters or even tissue-specific promoters can be used to drive transgene expression from the Rosa26 locus remained elusive. Therefore, we decided to test several ubiquitous and tissue-specific promoters in the modRosa26^LoxP^ locus in combination with rapid ES cell targeting by RMCE. Although the CMV, EF1α and pCAG promoters showed efficient and comparable transgene expression *in vitro* in ES cells, only the pCAG promoter induced high transgene levels *in vivo* in the modRosa26^LoxP^ locus. In contrast to the pCAG and EF1a promoters, the CMV promoter was not active in most tissues *in vivo*, consistent with other studies showing that the CMV promoter is susceptible to silencing effects in transgenic mice [40]. It is possible that the NeoR cassette interferes with the transgene expression, depending on the promoter used.

When targeted to the Rosa26 locus without shielding the Rosa26 promoter, the pCAG promoter showed mosaic expression in skeletal muscle, lung and liver [14]. Since it was suggested that the Rosa26 promoter can influence the expression of inserted transgenes [14], [17], we introduced a Stop cassette downstream of the Rosa26 sense promoter to shield our expression cassette. Indeed, transgenic ES cells with this modification showed no EGFP expression *in vitro* when no exogenous promoter was inserted, indicating that the Rosa26 promoter was functionally silenced. In the modRosa26^LoxP^ locus, the pCAG promoter showed strong and ubiquitous EGFP expression in skeletal muscle and other organs analyzed. Only in the liver the EGFP expression remained mosaic, showing a clear improvement to previous pCAG driven transgene expression from the Rosa26 locus [14]. Possibly, removing the NeoR cassette or improved shielding of the Rosa26 promoter elements could further improve ubiquitous transgene expression using the pCAG promoter targeted to the Rosa26 locus. A clear advantage of the pCAG promoter is that it yields very high expression levels. Thus, pCAG promoter mice enable tracing of live cells for differentiation studies, electrophysiology and intravital microscopy in which recombination events need to be monitored by direct EGFP fluorescence. This is in contrast to the endogenous, ubiquitously active Rosa26 promoter that results in low and often insufficient levels of transgene expression that is not detectable without further immunostaining [12], [13], [46].

Using Cre recombinase as a sensitive reporter that was monitored by ActB-EGFP reporter mice [15], we analysed specificity tissue-specific promoters in the modRosa26 locus. Although αSMA and VeCad promoters mostly retained their specificity *in vivo* when targeted to the modRosa26 locus, rare ectopic transgene expression was observed. Col1a1 promoters entirely lost the tissue-specificity, resulting in ubiquitous transgene expression. Although we could show that the endogenous Rosa26 promoter was functionally silenced in the modRosa26 locus, it is possible that inserted tissue-specific promoters lose specificity due to positional influences. Therefore, using the modRosa26 locus in combination with tissue-specific promoters driving Cre requires careful characterization. However, unspecific or mosaic Cre expression is a general problem when using isolated promoter constructs and can often only be circumvented by a knock-in of Cre into the endogenous gene locus. When high amounts of non-conditional transgene expression from tissue-specific promoters are required, classical transgenic mice with multiple transgene insertions are still inevitable despite all their disadvantages, as mentioned above.

In order to facilitate high level transgene expression in a tissue-specific manner along with the advantages of single-copy integration into a defined locus, we now generated the modRosa26^FRT^ locus. We combined Flp/FRT-mediated RMCE into the modRosa26^FRT^ locus with conditional Cre/LoxP-mediated transgene expression from the pCAG promoter. This approach allows for reliable high-level transgene expression in the desired tissue using established Cre lines. Using this approach we generated two reporter mouse lines. The great advantage of mR26CS-EGFP reporter mice is the high EGFP fluorescence that allows monitoring of recombination events in live cells. Going one step further, mR26CS-Luc reporter mice even allow monitoring of recombination events in live animals upon luciferase injection. In particular, this allows initial screening of new Cre lines without extensive histological analysis, since promising candidates can be selected very rapidly by Xenoimaging. In addition, these mice could be used to monitor tumor formation in combination with Cre/LoxP conditional deletion of tumor suppressors or activation of oncogenes.

In summary, we modified the Rosa26 locus to facilitate the use of exogenous promoters and to accelerate the generation of transgenic ES cells and transgenic mice for gain-of-function studies. We characterized several ubiquitous and tissue-specific promoters *in vivo*. Using tissue-specific promoters for low-level transgene expression or combined Flp-RMCE and Cre/LoxP conditional transgene expression for high-level expression, we accelerated the generation of single-copy transgenic mice targeted to a defined locus as an alternative to classical transgenic mice produced by pronuclear microinjections. When well-characterized Cre lines are available for the tissue of interest, we believe that our system is preferable to classical transgenic mice, since it offers reliable and predictable expression profiles and rapid generation and therefore minimized costs and effort.

## Supporting Information

Figure S1 **Immunohistochemistry for EGFP in mR26-pCAG-EGFP mice.** DAB staining for EGFP on paraffin sections from mR26-pCAG-EGFP mice show ubiquitous EGFP expression in kidney, brain, heart and lung. For the brain, exemplary sections of cerebellum and the hippocampal CA1 region are shown. The liver shows broad EGFP staining in liver arteries and bile ducts, but mosaic staining in hepatocytes. EGFP-negative hepatocytes are indicated by asterisks.
(TIF)
