# Supplementary figures and images for: A Modified RMCE-Compatible Rosa26 Locus for the Expression of Transgenes from Exogenous Promoters
Source: PLoS One. 2012 Jan 13;7(1):e30011. doi: 10.1371/journal.pone.0030011 (PMC3258265; doi:10.1371/journal.pone.0030011)

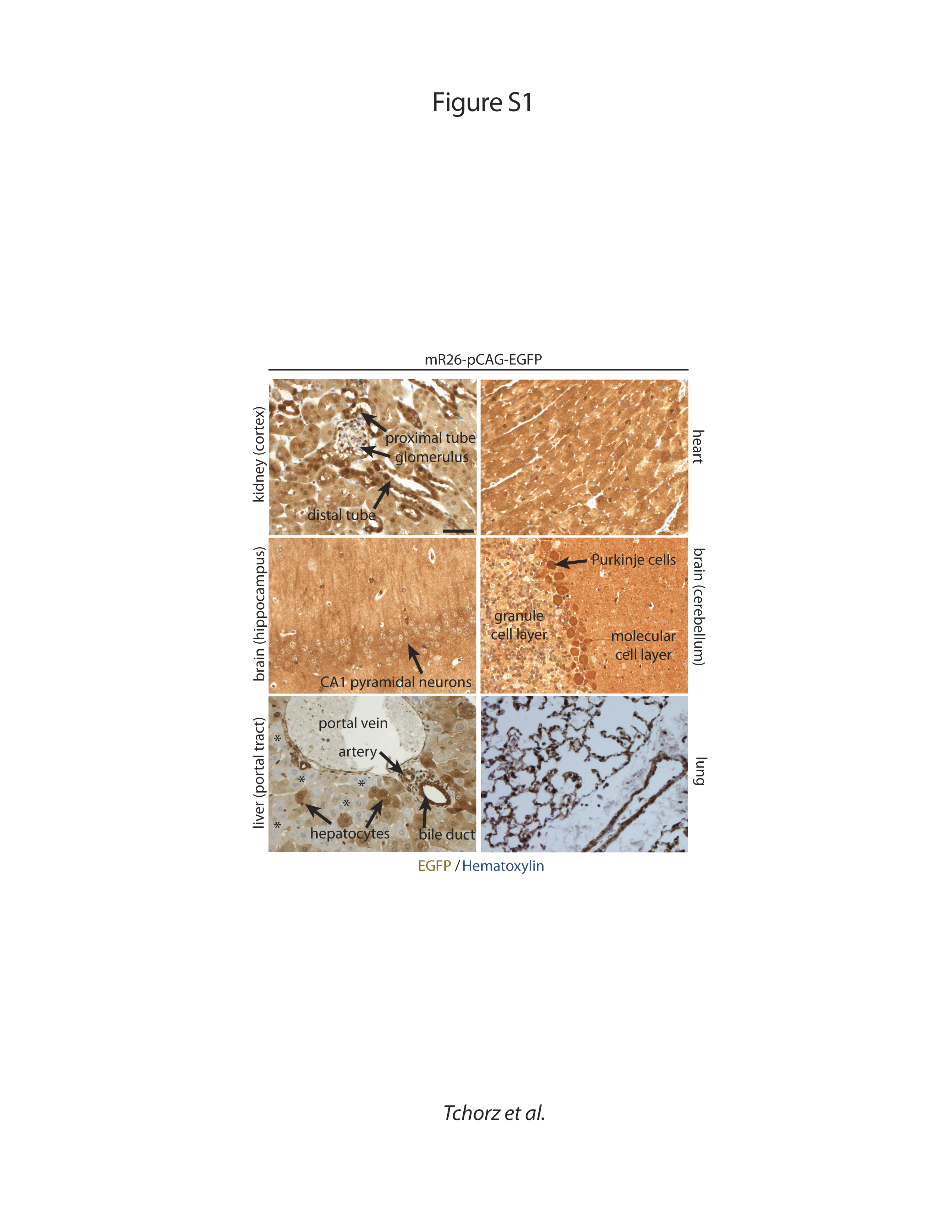

Supplement: Figure S1 — Immunohistochemistry for EGFP in mR26-pCAG-EGFP mice. DAB staining for EGFP on paraffin sections from mR26-pCAG-EGFP mice show ubiquitous EGFP expression in kidney, brain, heart and lung. For the brain, exemplary sections of cerebellum and the hippocampal CA1 region are shown. The liver shows broad EGFP staining in liver arteries and bile ducts, but mosaic staining in hepatocytes. EGFP-negative hepatocytes are indicated by asterisks. (TIF) [file pone.0030011.s001.tif]
